# Anti-Diabetic and Anti-Adipogenic Effect of Harmine in High-Fat-Diet-Induced Diabetes in Mice

**DOI:** 10.3390/life13081693

**Published:** 2023-08-05

**Authors:** Menna H. E. Morsy, Zohour I. Nabil, Samah T. Darwish, Rasha A. Al-Eisa, Amir E. Mehana

**Affiliations:** 1 Department of Zoology, Faculty of Science, Arish University, Arish 45511, Egypt; samaah_darwish@yahoo.com; 2 Department of Zoology, Faculty of Science, Suez Canal University, Ismailia 41522, Egypt; zohournabil@yahoo.com (Z.I.N.); amirelsayed@science.suez.edu.eg (A.E.M.); 3 Department of Biology, College of Sciences, Taif University, P.O. Box 11099, Taif 21944, Saudi Arabia; r.hasan@tu.edu.sa

**Keywords:** obesity, harmine, high-fat diet, diabetes

## Abstract

One of the most important health issues facing the world today is obesity. It is an important independent risk factor for developing type 2 diabetes. Harmine offers various pharmacological effects, such as anti-inflammatory and anti-tumor effects. The current study aims to investigate Harmine’s anti-diabetic and anti-adipogenic properties in albino mice after inducing low-grade inflammation with a high-fat diet (HFD). About forty-eight male albino mice were divided into four groups. Group 1: control mice were injected with daily saline and fed a normal chow diet of 21% protein for 5 months. Group 2: mice were treated daily with IP-injected Harmine (30 mg/kg body weight) and were fed a normal chow diet for 5 months. Group 3: mice were fed HFD to induce type 2 Diabetes Mellitus (T2DM) for 5 months. Group 4: mice were fed HFD for 14 weeks and treated with Harmine for the last 6 weeks. A figh-fat diet caused a significant increase in body and organ weight, lipid profiles, and destructive changes within the pancreas, kidney, and liver tissue. The administration of Harmine led to a remarkable improvement in the histological and ultrastructural changes induced by HFD. The findings indicate that mice cured using Harmine had lower oxidative stress, a higher total antioxidant capacity, and a reduced lipid profile compared to HFD mice. Harmine led to the hepatocytes partly restoring their ordinary configuration. Furthermore, it was noticed that the pathological incidence of damage in the structure of both the kidney and pancreas sections reduced in comparison with the diabetic group. Additional research will be required to fully understand Harmine and its preventive effects on the two forms of diabetes.

## 1. Introduction

Insulin resistance is a defective biologic response of target tissues, mainly the liver, muscle, and adipose tissue, to insulin stimulation [1]. Hyperinsulinemia and increased beta cell insulin production occur due to impaired glucose disposal brought on by insulin resistance [2]. The metabolic effects of insulin resistance can lead to visceral obesity, weight gain, hypertension, dyslipidemia, hyperglycemia, and dyslipidemia [3,4]. Type 2 diabetes can develop due to insulin resistance progression [4].

The great majority of diabetic patients (95%) have T2DM and are adults (20–79 years old) [5]. Obesity is the most frequent independent major risk factor for T2DM [6,7]. It is one of the world’s most serious health problems. A variety of endogenous or environmental factors can cause it. However, the most common causes are inequalities in food consumption, basic metabolism, and calories burned. Obesity could be defined as the accumulation of adipose tissue [8]. The body mass index (BMI) is used to refer to obesity. When the BMI exceeds 30 kg/m^2^, individuals are obese, but when it ranges from 25–30 kg/m^2^, they are overweight, not obese [9].

According to WHO, Saudi Arabia, Kuwait, the United Arab Emirates, and Bahrain are among the top ten countries regarding obesity rates, making Gulf countries the most obese in the world. Kuwait is the worst among the Gulf countries, with almost 42% of its population being obese. The obesity rates in Saudi Arabia and Qatar are 35.2% and 33.1%, respectively [10,11]. The expansion of industry, economic prosperity, improved lifestyles, and increased individual income are thought to be associated with a significant increase in obesity [12]. Furthermore, Egypt has the world’s 18th-highest obesity rate. Obesity-related deaths are thought to be around 115 thousand per year (19.08% of total estimated deaths in 2020) [13]. According to the “100 million health” study that was performed in Egypt in 2019 and that assessed 49.7 million mature Egyptians (18 years old), 39.8% of adults with a BMI of 30 kg/m^2^ or more were obese [14]. The islets of Langerhans are important in glucose regulation. The onset of hyperglycemia can prompt both insulin resistance and beta cell dysfunction. When beta cells become dysfunctional, insulin secretion decreases. The pancreas sends messages to the brain to enhance food intake (hunger), resulting in a higher insulin need and insulin resistance. Insulin-dependent tissues such as the liver, muscle, and adipose tissue begin to raise glucose levels by activating glycogenolysis, gluconeogenesis, and lipolysis, respectively. As a result, higher levels of non-esterified fatty acids (NEFAs) lead to higher levels of triglycerides, which can subsequently be converted into very-low-density lipoprotein (VLDL) or stored in the liver [15].

A hallmark of obesity is chronic low-grade inflammation and the altered secretion of adipokines, inflammatory cytokines, and hormones, including monocyte chemotactic protein-1 (MCP-1), C-reactive protein, IL-6, tumor necrosis factor-α (TNF-α), and adiponectin [16,17]. Additionally, this inflammation stimulates the growth of insulin resistance in muscle, liver, and adipose tissue [16,18,19].

Due to the abundance of bioactive phytochemicals found in medicinal plants, they play an important role in controlling diabetes [20]. Medicinal plants offer anti-diabetic characteristics that are reported to have no negative side effects. They have anti-diabetic chemicals such as flavonoids, alkaloids, phenolics, and tannins, which increase pancreatic tissue efficiency by raising insulin secretion [21]. Harmine is a natural source of β-carboline alkaloids that originates in a wide range of plants, particularly the harmal plant from the Middle East (*Peganum harmala*). Harmine exerts a broad spectrum of pharmacological actions owing to its anti-inflammatory, anticancer and anti-diabetic properties, and improves insulin sensitivity [22]. Harmine’s pharmacological activities include its capacity to effectively block monoamine oxidase (MAO). Serotonin, dopamine, norepinephrine, other neurotransmitters, and hormones are protected from destruction by MAO [23]. It is an effective dual specificity tyrosine-phosphorylation-regulated kinase 1 (DYRK1A) inhibitor with therapeutic promise regarding the treatment of diabetes in humans. It can induce beta-cell proliferation in vitro and in vivo [24,25].

Harmine inhibits the NF-κB signaling pathway, which allows it to suppress lipopolysaccharide-induced inflammatory responses in a RAW264 (a macrophage cell-line) inflammatory cell model. In addition, Harmine significantly reduced lung damage and the levels of cytokines that cause inflammation, such as IL-6, TNF-α, and IL-1, in a mouse model of severe lung damage [26].

This study evaluated Harmine’s anti-diabetic and anti-adipogenic properties in albino mice after inducing low-grade inflammation with a high-fat diet. Based on the investigated properties and pharmacological activities of this material, it was hypothesized that this effective material may be able to develop glycemic control and increase insulin sensitivity.

## 2. Materials and Methods

### 2.1. Experiments Preparations

#### 2.1.1. Preparation of Harmine

Harmine (C13H12N2O) was purchased from Sigma-Aldrich (CAS.NO. 442-51-3). For the preparation of 30 mg/kg of body weight of Harmine stock solution, 250 mg of Harmine was dissolved in 83.333 mL of saline (Sodium Chloride 0.9%). The stock solution was divided into aliquots of 2 mL Eppendorf tubes (approximately 41 Eppendorf tubes). All tubes were stored at −20 °C until use. The Harmine solution was heated to 42 °C and stirred in a water bath before use [27].

#### 2.1.2. Induction of T2DM by HFD

The composition of 1 kg of the HFD (60 kcal% from fat) was 50 g of cellulose, 204 g of corn starch, 336 g of casein, 4 g of methionine, 263 g of sheep fat, 100 g of corn oil, 34 g of vitamin mix and 9 g of mineral mix [28]. The induction of T2DM in mice was attained by feeding them with the HFD for 5 months.

### 2.2. Experimental Animals

About 48 healthy male albino mice weighing 16–20 g were used for this study. They were purchased from the Theodor Bilharz Research Institute, Giza, Egypt. The experimental male albino mice at the age of four weeks were maintained in well-aerated polyethylene cages (65 cm × 25 cm × 15 cm) at room temperature (25 ± 5 °C). They were exposed to a 12 h dark/12 h light cycle. They were provided with commercial food pellets containing standard nutrients obtained from Ibex International companies [29]. The Research Ethics Committee of the Suez Canal University Faculty of Science, Egypt, approved the experiment with code REC127/2022.

After the period of acclimation, the body weight, fasting blood glucose (FBG), intraperitoneal glucose tolerance test (ipGTT), and random blood glucose (RBG) of all animals were determined at the beginning of the study. Mice with 126–170 mg/dL fasting glucose levels were chosen for this study.

### 2.3. Experimental Design

There were 12 animals per group, divided as follows:

Group 1: Control group—mice were daily IP injected with saline and were fed a normal chow diet of 21% protein for 5 months.

Group 2: Harmine group—mice were daily IP injected with Harmine (30 mg/kg bodyweight) and were fed a normal chow diet for 5 months.

Group 3: HFD group—mice were fed with a HFD, as mentioned before, for 5 months to induce T2DM.

Group 4: HFD group treated with Harmine—mice were fed with a HFD for 14 weeks and IP injected with Harmine (30 mg/kg body weight) daily in the last 6 weeks.

### 2.4. Measured Parameters

#### 2.4.1. Intraperitoneal Glucose Tolerance Test (ipGTT)

At the experiment’s beginning, middle, and final week, ipGTTs were performed in all groups. Mice were transferred into clean cages without food and with full access to drinking water overnight. The next day, they were weighed before receiving an IP injection of the glucose dose (2 g per each kilogram of body weight). The glucose levels were then measured in the blood drawn from the tail vein at 0 (immediately before injection), 10, 20, 30, 60, and 120 min [30].

#### 2.4.2. Blood Glucose Levels

The random and fasted blood glucose levels were collected from the tail artery of the mice and then measured using a glucometer (Glucostar, MEDLAND CO, Middle East, and Africa). Results were expressed as mg/dL [31].

#### 2.4.3. Body Weights

The random and fasted weights of the mice were measured weekly in all groups during the experimental period using a digitally sensitive balance (3 digits).

#### 2.4.4. Relative Organ Body Weight

The weight of the liver, kidneys, pancreas, fats, and spleen was measured in grams. However, the relative weight of the organ body for each animal was calculated according to Kilany et al. [32], as follows:

$$\;\;\mathrm{Relative}\;\mathrm{organ}\;\mathrm{body}\;\mathrm{weight}\;=\frac{\mathrm{Absolute}\;\mathrm{organ}\;\mathrm{weight}\;\left(g\right)}{\mathrm{Body}\;\mathrm{weight}\;\mathrm{of}\;\mathrm{mouse}\;\mathrm{on}\;\mathrm{sacrifice}\;\mathrm{day}\;\left(g\right)\;}\;\times 100$$

#### 2.4.5. Body Mass Index (BMI)

At the end of the experiment, the mice were fasted overnight. They were weighed and anesthetized using diethyl ether. After anesthesia, their body length was measured from nose to anus in all groups to calculate the body mass index. The BMI was calculated using the formula shown below [32]:

BMI = g/cm^2^

where g is the mouse weight in grams, and cm^2^ is their height in centimeters squared.

### 2.5. Biochemical of Blood

#### 2.5.1. Blood Sampling

After mouse dissection, blood samples were collected via cardiac puncture under anesthesia for biochemical estimations. Some blood samples were collected in EDTA-containing tubes and then instantly cooled in a refrigerator at 6 °C for further glycated hemoglobin (HbA1c) analysis. The remaining blood was centrifuged at 3000 rpm for 10 min, then the serum was collected and stored at −20 °C for further analysis. The serum lipid profile, kidney function, liver function, glucose, insulin, lipid peroxidation, and total antioxidant capacity were measured.

#### 2.5.2. Blood Glucose Levels

Glucose was evaluated using a commercial diagnostic kit (SPINREACT, S.A. Ctra. Santa Coloma, Spain) and expressed in mg/dL [33].

#### 2.5.3. Determination of Insulin Levels

The serum insulin level was measured after coagulation at room temperature and after 10 min of centrifugation at 3000 rpm using an ultra-sensitive mouse ELISA Kit (SPINREACT, S.A. Ctra. Santa Coloma, Spain) [31].

#### 2.5.4. Determination of HbA1c

Blood samples were treated using tris hydroxyl methyl amino methane buffer to release hemoglobin from the red blood cells. A fraction of the blood sample was mixed with sodium lauryl sulfate (SLS) in a reaction chamber to obtain the SLS–hemoglobin complex. The amount of total hemoglobin was determined by observing the SLS–hemoglobin complex at a wavelength of 525 nm [34,35].

#### 2.5.5. Determination of Serum Alanine Aminotransferase Activity (ALT)

Using a commercially available kit from the SPINREACT organization (SPAIN, REF: SP41274), the serum ALT was measured [36].

#### 2.5.6. Determination of Serum Uric Acid and Creatinine Concentrations

The serum uric acid was determined using an available commercial kit (Ref: ab65344, USA) based on (Uricase/PAP) the enzymatic colorimetric technique [33]. The serum creatinine concentration was measured using a colorimetric–kinetic market kit from an Egyptian company, Diamond Diagnostics. The assay was based on Jaffe’s description of the interaction between creatinine and sodium picrate [37].

#### 2.5.7. Determination of Lipid Profile

A CHOD-PAP enzymatic reaction was used to calorimetrically assess the serum cholesterol. The kit was purchased from Biotechnology, S.A.E., Reference:230 001 [38]. Triglycerides in the blood were calorimetrically measured using the GPO-PAP enzyme [39]. The kit was purchased from Biotechnology, S.A.E., Reference: 314 001. Using a commercial kit, high-density lipoprotein (HDL) cholesterol levels in the blood were measured (Vitro Scient, Egypt, REF: 1581) [40]. Further, using a commercially available kit, the serum low-density lipoprotein (LDL) cholesterol was calculated (Vitro Scient, Egypt, REF: 1591) [40].

#### 2.5.8. Determination of Malondialdehyde (MDA) and Total Antioxidant Capacity Content in Serum

Using a kit from Biodiagnostics, CAT.NO. MD 25 29, the malondialdehyde (MDA) content was determined [41]. Using a commercial kit, the total antioxidant capacity concentration of the blood serum was determined (Bio diagnostics, Egypt, CAT.NO. TA 25 13) [42].

### 2.6. Histological Examination

The mice’s liver, kidney, and pancreas were rapidly removed. The selected organs were then rinsed with saline (0.9% NaCl) to remove the blood. They were then weighed, rinsed in ice-cold saline, and dried on filter paper before being preserved in 10% neutral buffered formalin overnight and treated in order to obtain paraffin slices that were 3–5 μm thick. Hematoxylin and eosin (H&E) stain was used to stain the sections for histological evaluation. This was performed using standard histology processing techniques [43].

### 2.7. Morphometric Study

The histological sections of pancreatic islets in all groups of the experiment were photographed using a light binocular microscope (Leica DM 1000). To measure these morphometric parameters, images of the histological sections of three animals for each group were taken; from each animal, eight slides representing the whole pancreas were taken to measure these morphometric parameters. Twenty-four histological sections from each group were obtained using a 40× objective lens and analyzed using ImageJ software to measure the macroscopic area of the islets. Briefly, the pancreatic islets were surrounded using the free-hand tool to draw an oval shape exactly surrounding the islets in order to measure the area (μm^2^). After that, the major diameter was estimated by measuring the length of the previously drawn oval shape surrounding the islets from one pole to the other. The minor diameter was estimated by measuring the widest length of the same shape in the examined islets [44].

### 2.8. Electron Microscopy Analysis

Small sections of pancreas and liver were removed and placed in 0.1 M Na–cacodylate buffer for 24 h with 2% glutaraldehyde fixative, followed by preservation with 1% osmium tetra-oxide, and rinsed in 0.1 M phosphate buffer at 4 °C. Following this, the tissues were gradually dehydrated in ethyl alcohol, and they were subsequently infiltrated with resin. Using a Leica Ultracut UCT, Heidelberg, Germany, ultramicrotome, tissue blocks were cut into semi-thin sections; they were then stained with toluidine blue and examined using a light microscope to determine their general orientation and the areas that needed to be selected for an electron microscope examination [45]. Ultra-thin sections were cut using the ultramicrotome and stained with a mixture of uranyl acetate and lead citrate. The stained ultra-thin sections were examined and photographed using (TEM-JEOL JEM-2100) in the electron microscopy unit at Mansoura University [46,47].

### 2.9. Statistical Analysis

Software called GraphPad Prism (Version 8) was used for statistical analysis. To determine the statistical significance of differences between the tested groups, a one-way analysis of variance (ANOVA) test was conducted, and the Tukey test was used as a post hoc analysis. A significant difference occurs when the *p*-value is less than 0.05. The values were expressed as mean ± standard error (SE) [48].

## 3. Results

### 3.1. Characterization of Diabetic Animal Mode

#### 3.1.1. Body Weight

**Figure 1:**
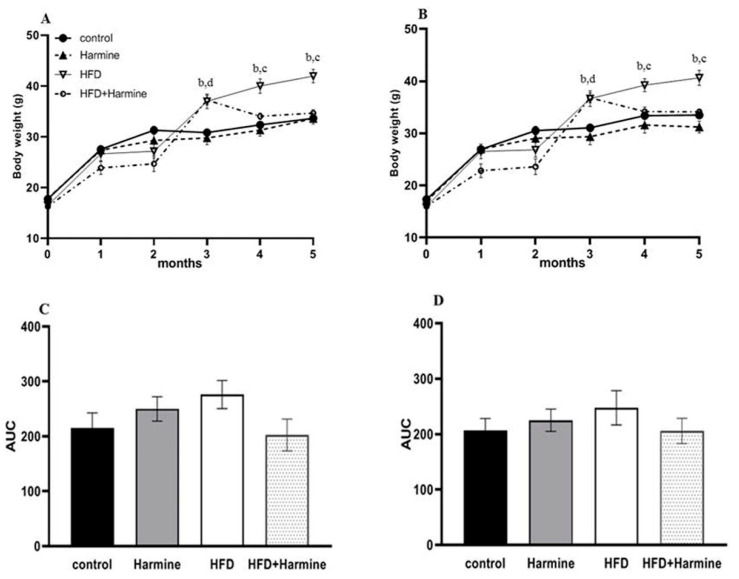
Body weights of mice during 5 months of the HFD experiment. (**A**) represents the random body weight and (**B**) expresses the fasted body weight, (**C**) describes the AUC of graph A, and (**D**) describes the AUC of graph B. Data offered as mean ± SEM (n = 9). Data were statistically analyzed using one-way ANOVA followed by the post hoc Tukey test. The letters b and d indicate a significant difference between the control group and HFD, and between the control group and HFD + Harmine group, respectively. The letter c reveals a significant difference between the HFD group and HFD + Harmine group.

There was a significant increase in the random and fasted body weights of the HFD group starting from the third month until the fifth month (post hoc Tukey test control vs. HFD; HFD vs. HFD + Harmine *p* = 0.0109, *p* = 0.0118 for the fourth month; *p* = 0.0012, *p* < 0.0001 for the fifth month; respectively). The area under the curve (AUC) was measured for the random and fasted body weights. The random and fasting body weights did not significantly differ across groups in the AUC (Figure 1).

#### 3.1.2. Body Mass Index and Relative Organ Weight

**Figure 2:**
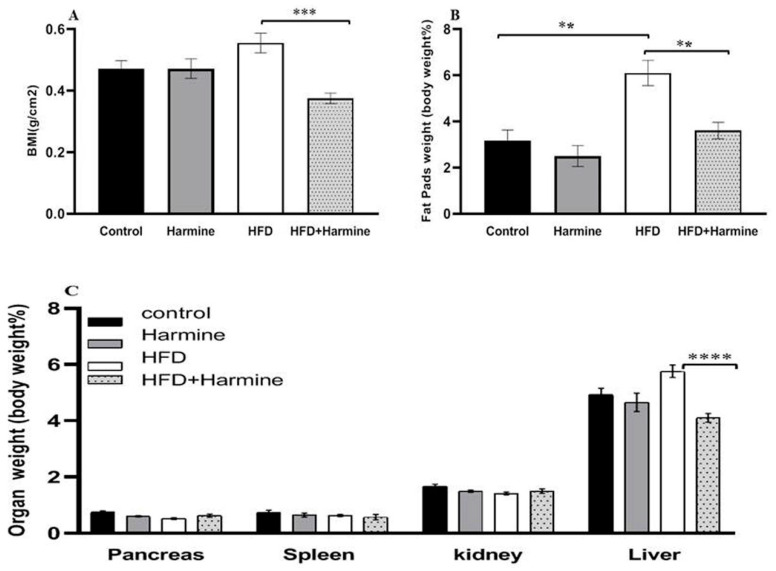
Effect of Harmine on the body mass index and relative organ weights in the control and experimental groups of mice at the end of the HFD-induced diabetes experiment. (**A**) Body mass index (BMI). (**B**) The relative weight of fat pads as % of body weight. (**C**) The relative weight of organs (pancreas, spleen, kidney, and liver) as % of body weight. Data are obtainable as mean ± SEM (n = 6). Asterisks (**, ***, and ****) indicate a significant difference with *p* < 0.01, *p* < 0.001, and *p* < 0.0001, respectively.

The relative organ weights and body mass index in the control and several treatment groups are shown in Figure 2. There was an overall significant difference (ANOVA: F = 8.347, *p* = 0.0006). The data of the HFD group showed a significant increase in the body mass index compared to the HFD + Harmine group (*p* = 0.0002). The statistical analysis of the relative weight of the liver in the HFD group was significantly higher than the HFD + Harmine group (post hoc Tukey test; HFD vs. HFD + Harmine: *p* < 0.0001). On the other hand, the amount of fat accumulating around the gonads and in the peritoneum significantly increased in the HFD mice in contrast to the control and HFD + Harmine groups (post hoc Tukey test; control vs. HFD; HFD vs. HFD vs. HFD + Harmine: *p* = 0.0013, *p* = 0.0047, respectively).

#### 3.1.3. Blood Glucose Levels

**Figure 3:**
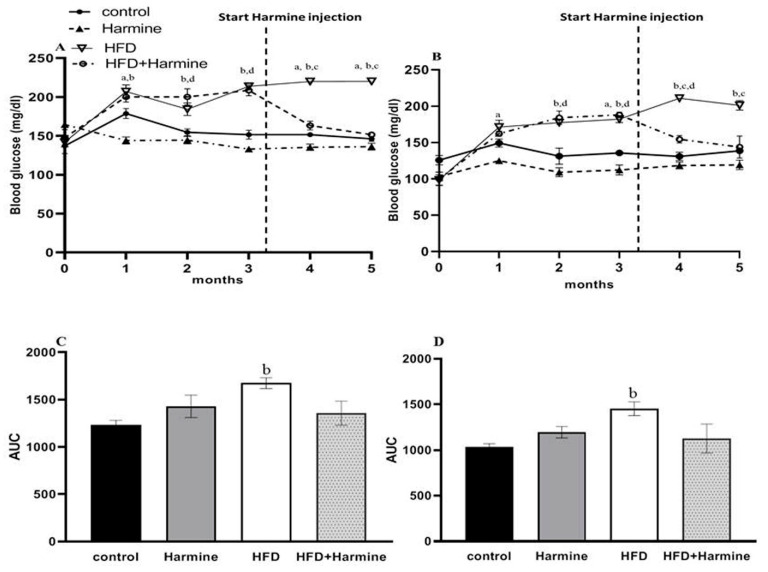
Blood glucose levels of mice throughout the HFD experiment. (**A**) Represents the random blood glucose over 5 months, (**B**) represents the fasted blood glucose, (**C**) displays the AUC of graph A, and (**D**) represents the AUC of graph B. Data obtainable as mean ± SEM (n = 9). One-way ANOVA was used to statistically analyze the data, and the Tukey test was then performed. The letters a, b, and d indicate a significant difference between the control group and Harmine group; the control group and HFD group; and the control group and HFD + Harmine group, respectively. The letter c indicates a significant difference between the HFD group and HFD + Harmine group.

The random and fasted blood glucose levels are shown in Figure 3. There was an overall significant difference in the random blood glucose (ANOVA: F = 6.779, *p* = 0.0128) and fasted blood glucose (ANOVA: F = 4.918, *p* = 0.0102) between the groups during the experiment. In the random blood glucose, the HFD group’s blood glucose levels were significantly elevated compared to the control group. The fasted blood glucose level results showed a significant difference between the control and HFD groups from the second month until the end of the experiment. In the fourth month, there was a significant decrease in the blood glucose levels in the HFD group treated with Harmine compared to the HFD group. The AUC analysis outcome showed a significant increase in the HFD group compared to the control in terms of the fasted and random blood glucose (*p* = 0.0347 and *p* = 0.0267, respectively).

#### 3.1.4. Intraperitoneal Glucose Tolerance Test (ipGTT)

**Figure 4:**
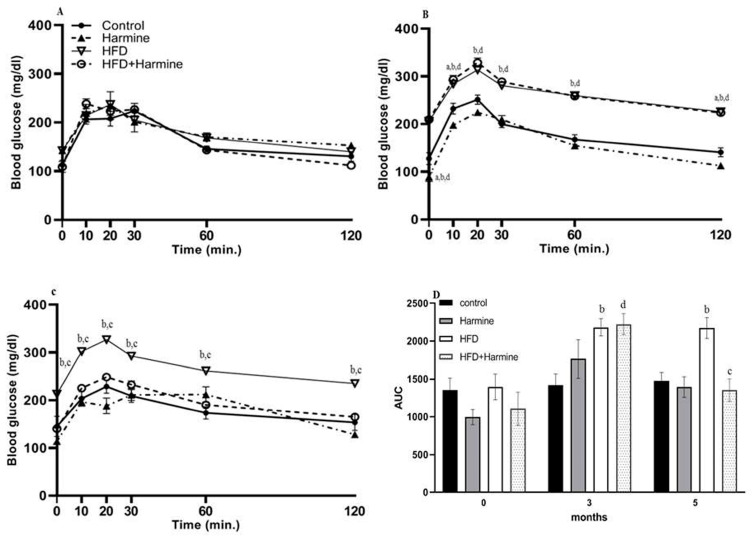
Effect of Harmine on the intraperitoneal glucose tolerance test (ipGTT) performed at 0, 3, and 5 months during the HFD-induced diabetes experiment. (**A**) Represents the ipGTT at month 0, (**B**) represents the ipGTT at month 3, (**C**) represents the ipGTT at month 5, and (**D**) shows the AUC of all ipGTTs. Data offered as mean ± SEM (n = 9). The letters a, b, and d indicate a difference between the control group and Harmine group; control mice and HFD group; and control mice and HFD + Harmine group, respectively. The letter c indicates a significant difference between the HFD group and HFD + Harmine group.

Intraperitoneal glucose tolerance tests (ipGTTs) after 0, 3, and 5 months in the control and treated groups revealed that all groups exhibited normal and identical time courses of blood glucose tolerance at month 0. In contrast, at month 3, there was an overall significant difference between groups (F = 88.08, *p* < 0.0001). Within the fifth month of treatment, the groups significantly differed (F = 50.31, *p* = 0.0002). The area under the curve was determined for all ipGTTs. The AUC analysis at month 0 exhibited no significant difference between all groups. However, in the fifth month, there was a significant increase in the HFD group compared with the control group (*p* = 0.0079), and a significant decrease in the HFD + Harmine group when compared to the HFD group (*p* = 0.0019) (Figure 4).

### 3.2. Biochemical Parameters

#### 3.2.1. ALT Activity, Creatinine, and Uric Acid Concentrations

**Figure 5:**
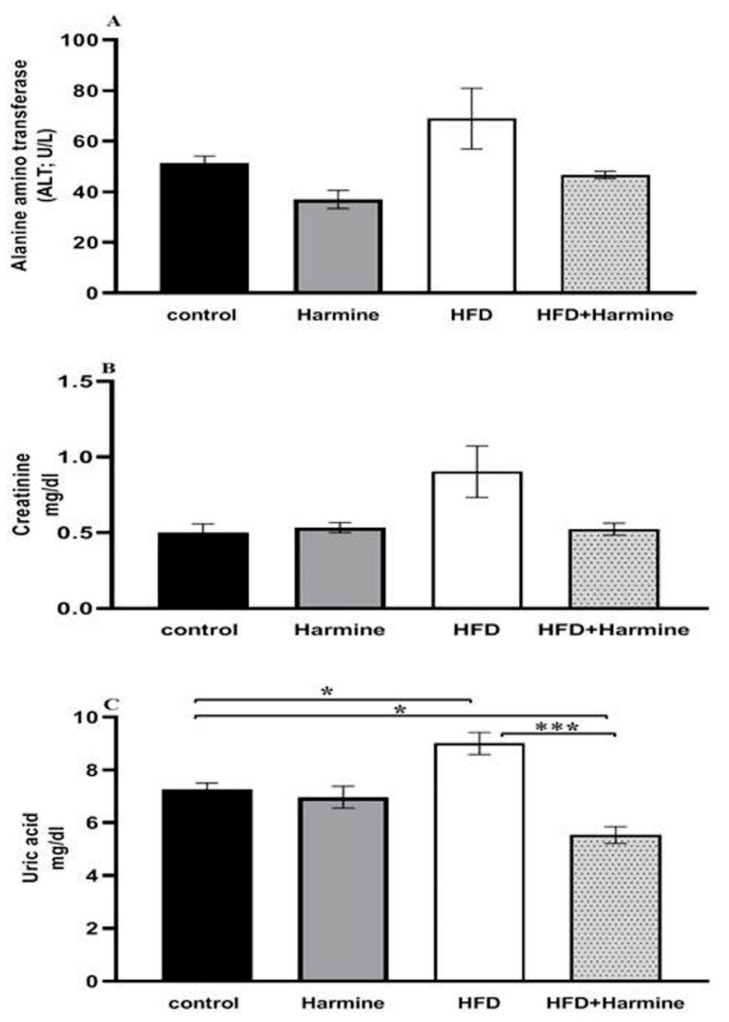
Effect of Harmine on alanine aminotransferase (ALT) activity (**A**), serum creatinine (**B**), and uric acid (**C**) at the end of the HFD experiment. Values are exhibited as means ± SEM (n = 6). Asterisks (* and ***) indicate a significant difference with *p* < 0.05 and *p* < 0.001, respectively.

There were no appreciable variations in the ALT activity between the HFD and control group, and the HFD and HFD + Harmine group. Moreover, there were no significant differences between the HFD and other treated groups in terms of creatinine. The results of the uric acid level in the HFD group showed a significant increase between other groups (post hoc Tukey test; control vs. HFD, control vs. HFD + Harmine, and HFD vs. HFD + Harmine with *p* = 0.0346, *p* = 0.0349 and *p* = 0.0006, respectively) (Figure 5).

#### 3.2.2. Blood Glucose Levels, Glycated Hemoglobin A1C, and Insulin

**Figure 6:**
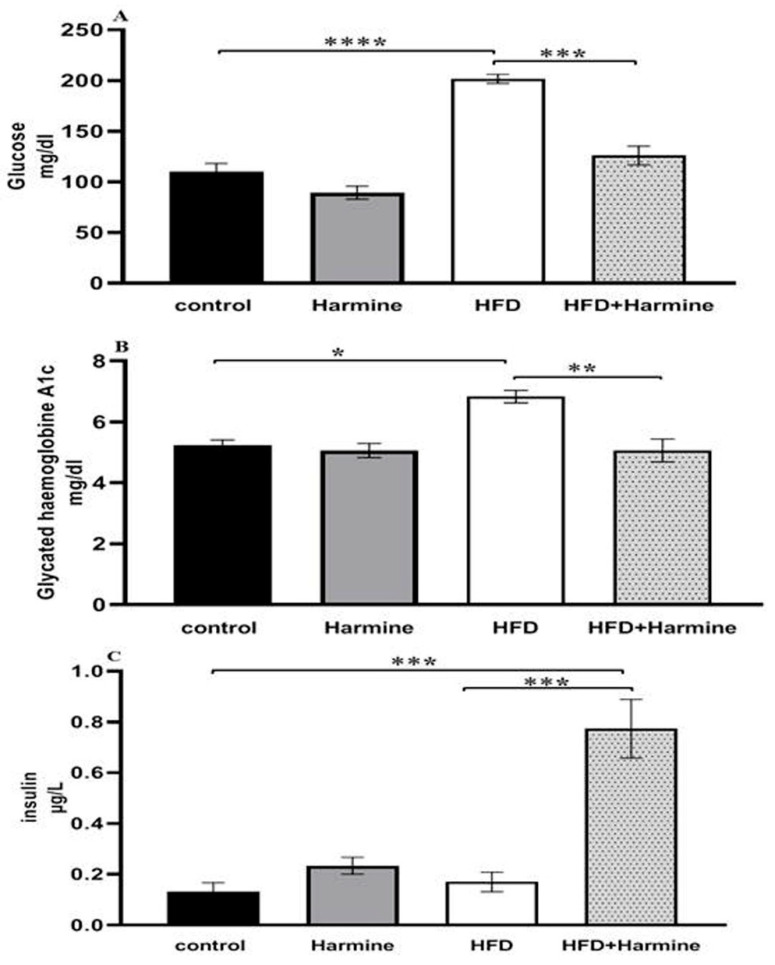
Levels of blood glucose (**A**), glycated hemoglobin A1c (**B**), and insulin (**C**) in control and experimental groups at the end of the HFD experiment. Values are proven as means ± SEM (n = 6). Asterisks (*, **, *** and ****) indicate a significant difference with *p* < 0.05, *p* < 0.01, *p* < 0.001 and *p* < 0.0001, respectively.

The HFD group exhibited diabetic values in both glucose and HbA1c when compared to the control group. Moreover, there was a significant decrease in the blood glucose levels of the HFD group that received the Harmine compared to the HFD mice (post hoc Tukey test; control vs. HFD, HFD vs. HFD + Harmine with *p* < 0.0001 and *p* = 0.0003, respectively). There was a significant increase in the HFD group treated with Harmine when compared to the HFD and control groups in terms of the insulin concentration (post hoc Tukey test; control vs. HFD, control vs. HFD + Harmine, HFD vs. HFD + Harmine with *p* = 0.9773, *p* = 0.0005, and *p* = 0.0008, respectively) (Figure 6).

#### 3.2.3. Lipid Profile

**Figure 7:**
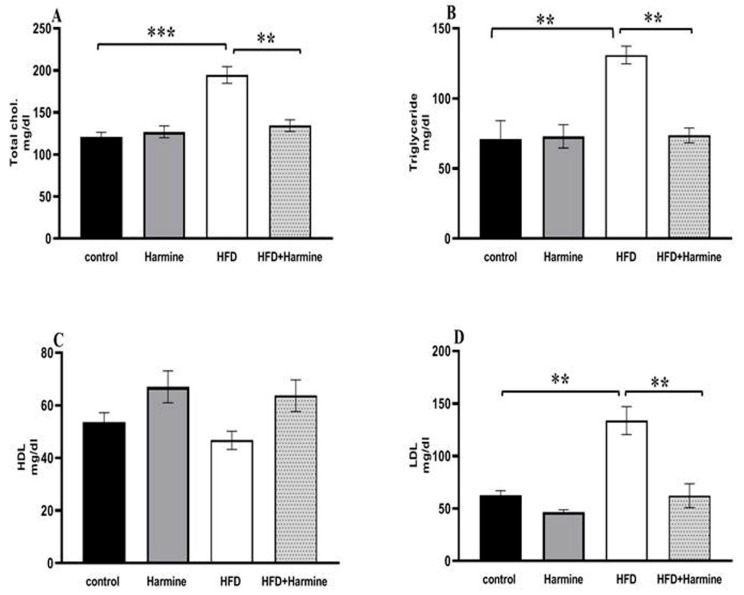
Effect of Harmine on serum lipid profile in the control and experimental groups at the end of the HFD experiment. (**A**) Total cholesterol, (**B**) triglycerides, (**C**) HDL, and (**D**) LDL. Values are displayed as means ± SEM (n = 6). Asterisks (** and ***) indicate a significant difference with *p* < 0.05 and *p* < 0.001, respectively.

Compared to the control group, the HFD group had significantly higher serum levels of triglycerides, cholesterol, and LDL. On the other hand, no difference was found between the groups in terms of the HDL levels (Figure 7).

#### 3.2.4. Malondialdehyde and Total Antioxidant Capacity

**Figure 8:**
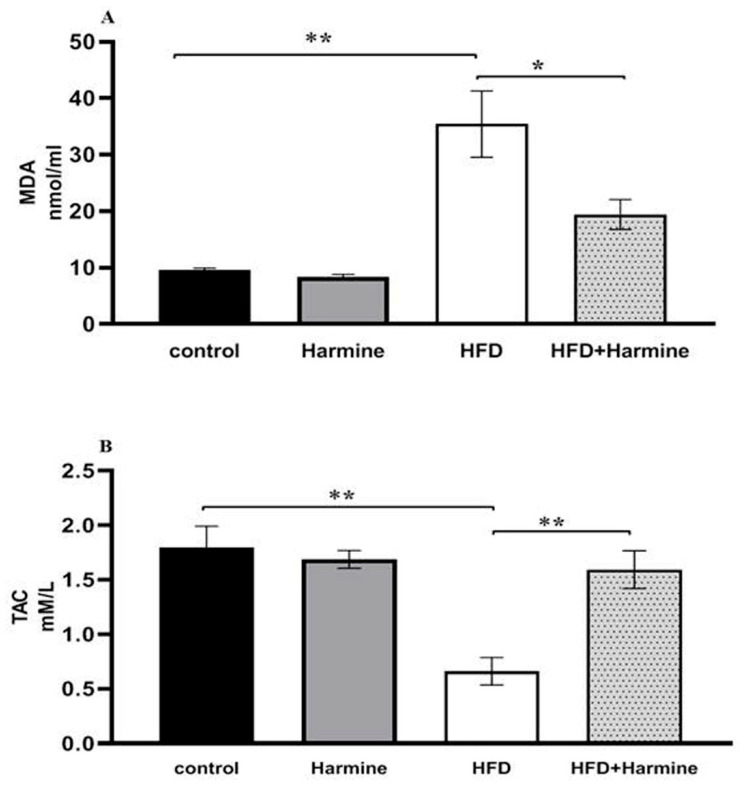
(**A**) Effect of Harmine on MDA and the (**B**) total antioxidant capacity (TAC) at the end of the HFD experiment. Values are revealed as means ± SEM (n = 6). Asterisks (* and **) indicate a significant difference with *p* < 0.05 and *p* < 0.01, respectively.

The MDA content in the HFD group significantly increased compared to other groups. Additionally, the results of the total antioxidant capacity levels in the HFD group showed a significant decrease compared to the other groups (Figure 8).

### 3.3. Histological Studies

#### 3.3.1. Liver

**Figure 9:**
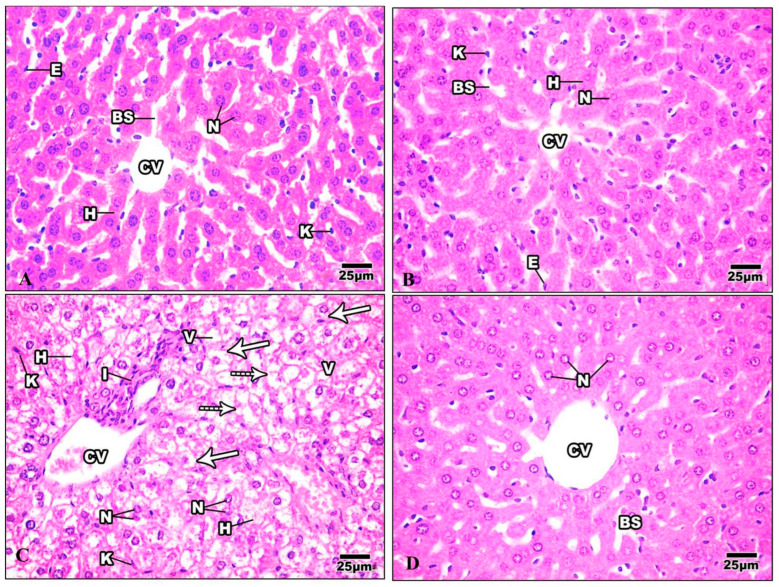
Photomicrographs of liver sections of the control group and other treated groups. (**A**) photograph of the liver section of the control group showing hepatic lobular parenchyma of normal mice, which reveals polyhedral hepatocytes (H) with spherical or oval nuclei (N), blood sinusoids (BS) lined by endothelial cells (E), and Von Kupffer cells (K). (**B**) Liver section of Harmine group showing hepatic lobular parenchyma of a mouse. Polyhedral hepatocytes (H) were shown to have spherical or oval nuclei (N) and blood sinusoids (BS), which were lined by endothelial cells (E) and Von Kupffer cells (K). The central vein can be seen (CV). (**C**) The liver section of the HFD group shows foci containing inflammatory cells (I) surrounded by empty-looking areas devoid of hepatocytes. Also, the presence of karyolytic and irregularly shaped nuclei (N) can be observed, as well as the presence of damaged hepatocytes (H). Enlarged Von Kupffer cells (K), micro steatosis (arrows), and macro steatosis (dotted arrows) can be observed. V: cytoplasmic vacuolization; CV: central vein. (**D**) The liver section of the HFD mice was cured using Harmine, and hepatocytes with similar features to the control group can be seen, with mild blood sinusoidal dilatation (BS) around the central vein (CV). Some hepatocytes show the margination of the nuclear chromatin (N). (H & E; 400×).

H&E-stained liver sections in the control group revealed a well-maintained hepatic lobular array with a well-preserved central vein architecture at the center, and radiating cords of hepatocytes were seen. The hepatocytes had a polyhedral shape and a granulated cytoplasm with tiny, homogeneous nuclei. Thin plates of these cells were spaced apart by large hepatic sinusoids, through which blood flows (Figure 9A). The hepatocytes of mice injected with Harmine appeared with spherical ovoid nuclei. Hepatic sinusoids filled the spaces between the plates and converged toward the central vein, lined by endothelial cells (Figure 9B). Liver sections of the HFD group showed variable histopathological changes, including distortion of the hepatic cellular architectures. Occurrences of cellular necrosis were observed in the liver of the HFD mice, as well as a big focal zone of injured hepatocytes, including numerous inflammatory cells. Unusually, large amounts of fat characterized widespread steatosis within the hepatocyte. They were greatly swollen and oval. It is possible to see a dilated, congested central vein exhibiting obvious endothelial lining degradation (Figure 9C). When compared to the HFD group, the liver’s histological structure in most diabetic mice treated with Harmine showed little degenerative changes. Hepatocyte strands partially reverted to their original structure, showing that the Harmine treatments enhanced recovery from HFD-induced liver injury. They showed good organization and a slight sinusoidal dilatation around the main vein (Figure 9D).

#### 3.3.2. Kidney

**Figure 10:**
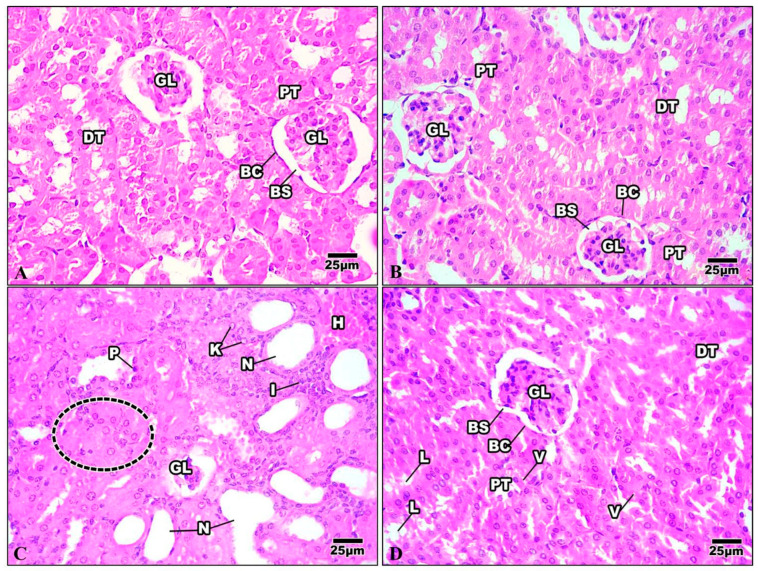
Photomicrographs of kidney sections of the control group and other treated groups. (**A**) Control kidney section showing the convoluted tubules and the regular organization of the renal corpuscles. Bowman’s capsule (BC) encloses the glomerulus (GL), with Bowman’s space (BS) separating the two. Columnar epithelial cells with a granular cytoplasm with large and round nuclei border the proximal convoluted tubules (PT). The distal convoluted tubules (DT) have wider lumina and are surrounded by cuboidal epithelial cells with a clear cytoplasm and spherical nuclei. (**B**) Control kidney section showing the convoluted tubules and the regular organization of the renal corpuscles. Bowman’s capsule (BC) encloses the glomerulus (GL), with Bowman’s space (BS) separating the two. Columnar epithelial cells with a granular cytoplasm with large and round nuclei border the proximal convoluted tubules (PT). The distal convoluted tubules (DT) have wider lumina and are surrounded by cuboidal epithelial cells with a clear cytoplasm and spherical nuclei. (**C**) Kidney section of the HFD group revealing shrinkage of the glomerulus (GL) with the loss of their nuclei and the occurrence of a dense eosinophilic mesangial matrix. The renal tubules suffer from acute tubular necrosis (N) and exhibit wide lumina with bleeding in some epithelial cells (H). Note that there is cellular swelling (dotted area), nuclear pyknosis (P), karyolysis (K), and some inflammatory cells (I). (**D**) The kidney section of the HFD group treated with Harmine revealed that the renal tubules restored their nuclei. However, treatment with Harmine showed few cytoplasmic vacuolizations (V) in the tubular lining epithelial cell, with some cell debris in the lumina (L) seen. Bowman’s capsule (BC) encloses the glomerulus (GL), with Bowman’s space (BS) separating the two. The apical brush borders of the columnar epithelial cells that line the proximal convoluted tubules (PT) are distinct. The distal convoluted tubules (DT), which have cuboidal epithelial cells with spherical nuclei and a clear cytoplasm, have wider lumina (H & E; 400×).

The Bowman’s capsule that encloses the glomerulus was evident in the kidney tissue of the control mice. A tuft of glomerular capillaries encloses the Bowman’s capsule, which is separated from it by the Bowman’s space. The renal cortex is the outer layer of the kidney tissue. Proximal convoluted tubules (PT) are numerous and have relatively small lumina; the distal ones are fewer and possess wider lumina. The distal convoluted tubules (DT) are lined by cuboidal epithelia with spherical nuclei and the transparent cytoplasm (Figure 10A). The kidney of mice injected with Harmine demonstrated a regular glomerulus structure surrounded by the Bowman’s capsule. The distal convoluted tubules are fewer in number and have broader lumina. The proximal convoluted tubules have minor lumina with a granular cytoplasm and large spherical nuclei (Figure 10B). The kidney of the HFD group showed that most of the renal tubules suffered from a severe degree of cellular damage, cytoplasmic deterioration, cytoplasmic vacuolization, nuclear pyknosis, and karyolysis. The renal tubules suffered from acute tubular necrosis marked by a loss of their nuclei or damage to most of their lining epithelial cells. Intensively damaged tubular lining epithelial cells were represented by the remnants of cytoplasmic materials and scattered cellular debris within the tubular lumina of the cortical tubules. Inflammatory cells can be seen in the intratubular spaces (Figure 10C). The damage caused to the kidney structure of the high-fat-diet group treated with Harmine was reduced compared to the HFD group. A few cytoplasmic vacuolizations in the tubular lining epithelial cells with a some cell debris in the lumina can be seen (Figure 10D).

#### 3.3.3. Pancreas

**Figure 11:**
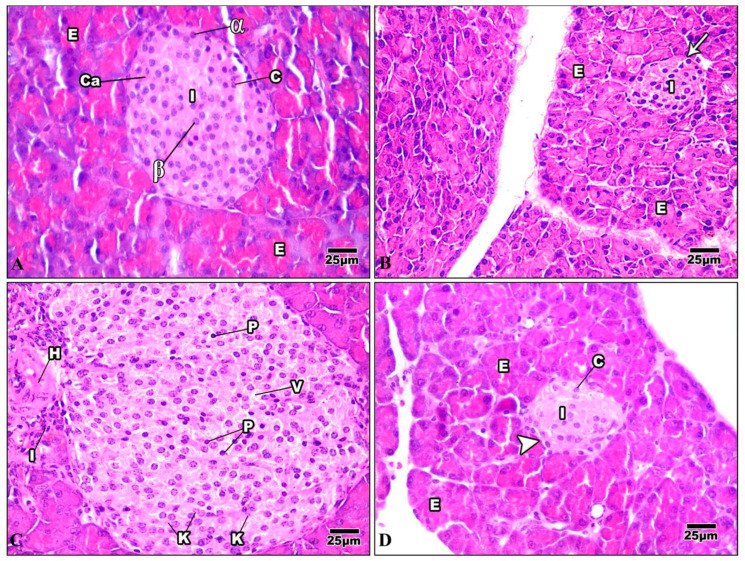
Photomicrographs of pancreatic sections of the control group and other treated groups. (**A**) A pancreatic section from the control group displays the typical construction of the pancreas. The exocrine parts (E) have islets of Langerhans (I) implanted inside them and arranged into small lobules with a broad basal portion and a narrow apical surface. The acinar cells have basal nuclei, a basophilic cytoplasm from the basal part, and an acidophilic cytoplasm from the apical portion. Cells of the islet are organized in cords (c) detached by blood capillaries (Ca). Note that the pyramidal cells that comprise the acini have basal nuclei and an apical acidophilic cytoplasm. The cells on the periphery are primarily α-cells (α) (which appear big), whereas most of the cells in the core are beta cells (β) (which appear smaller). (**B**) A pancreatic section from the Harmine group displays closely packed pancreatic acini lobules. The acini comprise pyramidal cells with basal nuclei and an acidophilic cytoplasm at their apex. Fenestrated capillaries are richly vascularized islets of Langerhans (I) with a regular outline (arrow) around each islet. They are inserted within the acinar cells (E) with a pale stained area. (**C**) In a pancreatic section of the HFD group, the hypertrophy of the islets can be seen, with a wide-ranging loss of architecture. Pyknotic nuclei (P) in certain cells are darkly stained, while karyolytic nuclei are seen in other cells (k). Cytoplasmic vacuolization (v) in the cells of the islet, frequently in the central part, can be seen. Note that inflammatory cells (I) and hemorrhage (H) adjacent to the islet can be noticed. (**D**) A pancreatic section of the HFD group treated with Harmine can be seen, displaying an islet of Langerhans (I) with a light stain that is encircled by intensely stained exocrine pancreatic acini (E). Islets to have well-defined boundaries (arrowhead) and formed cords of cells (c) (H & E; 400×).

The pancreas comprises exocrine cells called “acinar cells” and a simple epithelium. Each pancreatic acinus is surrounded by pyramidal acinar cells with a broad basal portion and a narrow apical surface. The endocrine pancreas is composed of small islands of endocrine cells. The islands are known as the islets of Langerhans (Figure 11A). The pancreas of mice injected with Harmine had both exocrine and endocrine components, and was nearly a normal pancreas. The acinar cells were pyramidal, their nuclei were basal, and their cytoplasm was basophilic from the basal part. The islets of Langerhans were light-staining spherical collections of endocrine cells spread among the acini. Most frequently, beta cells were seen in the center of the islet (Figure 11B). The HFD pancreatic tissue revealed pathological changes in both the exocrine and endocrine components. The islets showed distorted architecture; some appeared to show hypertrophy, while others showed shrunken islets. Signs of necrosis and beta cell destruction can be noticed, cytoplasmic vacuolization is evident, some cells showed darkly stained pyknotic nuclei, others showed karyolytic nuclei, and some inflammatory cells were evident (Figure 11C). After treatment with Harmine, the pancreas returned to a regular pancreas arrangement with a less pathological appearance than in the high-fat-diet mice. The islets of Langerhans appeared with normal structures and well-defined boundaries. They seemed to resemble big, pale-staining cells encased in basophilic, highly stained pancreatic acini (Figure 11D).

### 3.4. Morphometric Observations of Pancreatic Islets

**Figure 12:**
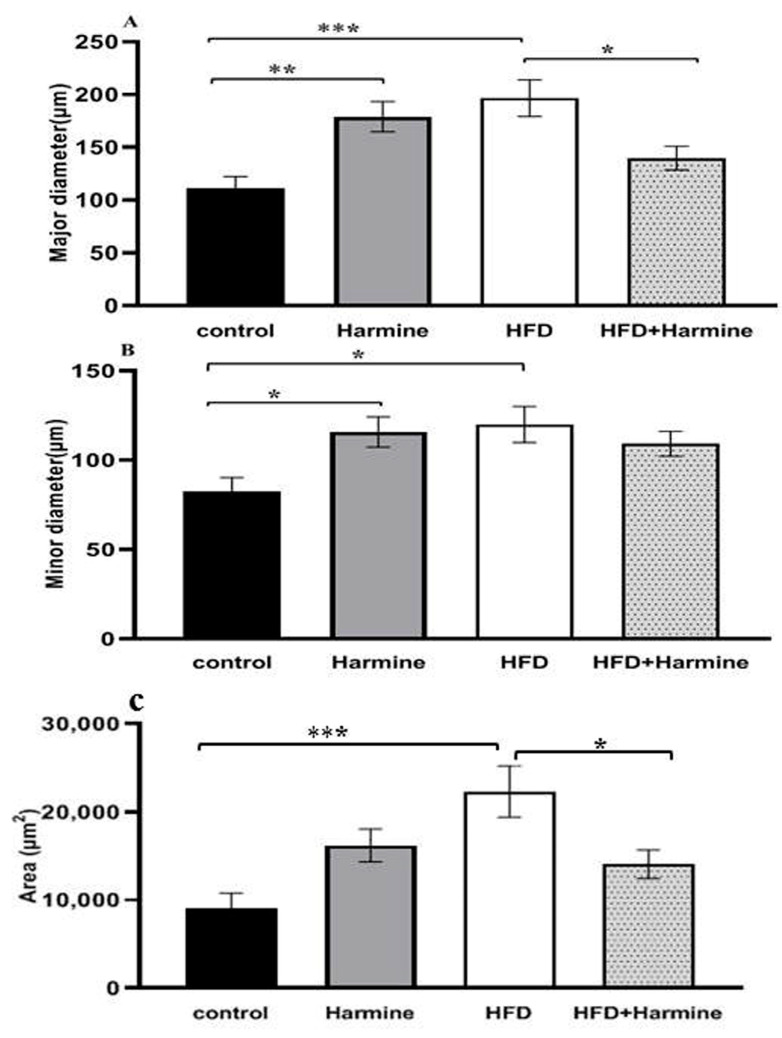
Morphometric analysis of the pancreatic islets from the HFD-induced diabetes experiment. Values are offered as mean ± SEM (n = 3). (**A**) Major diameter, (**B**) minor diameter, and (**C**) the area of pancreatic islets. Asterisks (*, **, and ***) indicate a significant difference with *p* < 0.05, *p* < 0.01, and *p* < 0.001, respectively.

There was a significant increase and hypertrophy in the diameter and area of pancreatic islets in the HFD group compared to the control group. These morphometric parameters were significantly decreased in the HFD + Harmine group (Figure 12).

### 3.5. Ultrastructural Studies

#### 3.5.1. Liver

**Figure 13:**
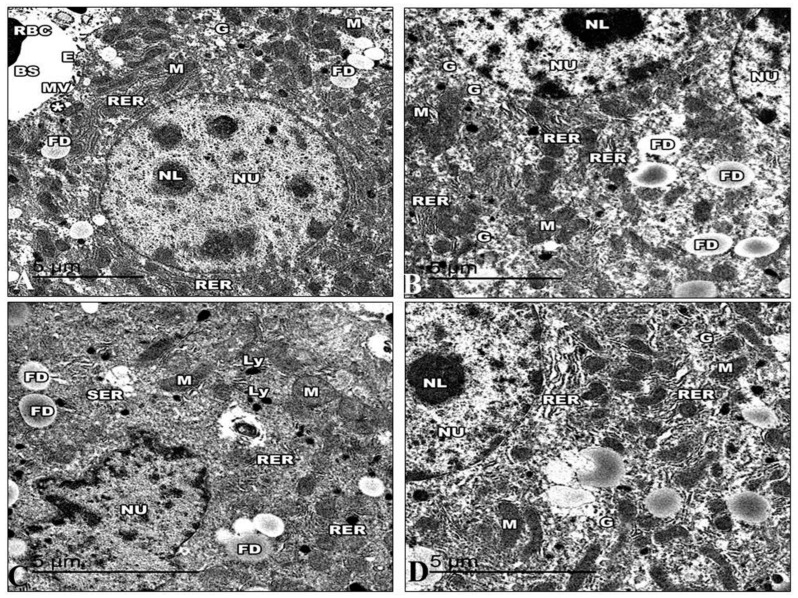
Electron micrographs of the liver sections of the control group and other groups. (**A**) An electron micrograph of a control group mouse’s liver, exposing the hepatic blood sinusoid of mice (BS). The hepatic cell membrane shows numerous microvilli (MV) extending into the Disse spaces (*). The blood sinusoids reveal fenestrated endothelia with small pores (E) and red blood cells (RBC). The hepatic cells show a rough endoplasmic reticulum (RER), areas of glycogen storage (G), fat droplets (FD), and numerous small mitochondria (M) with heavy electron-dense matrices. (**B**) An electron micrograph of the liver section of the Harmine group, exhibiting hepatocytes with the cytoplasm, containing binucleated hepatic cells with oval nuclei (NU) that have regular nuclear envelopes with well-defined nucleoli (NL). Rough endoplasmic reticulum (RER) arranged in groups of parallel cisternae, areas of glycogen storage (G), fat droplets (FD), and small, numerous mitochondria (M) with heavy electron-dense matrices. (**C**) An electron micrograph of the liver section of the HFD mice revealed hepatic cell nuclei (NU) with irregular nuclear envelopes and a decline in their heterochromatin. Large abundant fat droplets (FD) and lysosomes (Ly). A fragmented rough endoplasmic reticulum (RER), a vesiculated smooth endoplasmic reticulum (SER), and swollen mitochondria (M) losing their cristae can be detected. Note the decrease in glycogen deposits compared to the control mice. (**D**) An electron micrograph of the liver section of the HFD mice treated with Harmine illustrates hepatocytes with cytoplasm containing a nucleus (NU) and a distinct regular nuclear envelope. The nucleus has well-defined nucleoli (NL) and a nucleoplasm with euchromatin and heterochromatin. Mitochondria (M) in a rounded conformation can be seen. The rough endoplasmic reticulum (RER) is a parallel cistern close to the nuclear envelope. Areas of glycogen storage (G) are scattered into the cytoplasm. ((**A**); Magnification 10,000×. (**B**); Magnification 12,000×. (**C**); Magnification 15,000×. (**D**); Magnification 15,000×.).

The ultrastructure of the liver of the control mice is revealed in Figure 13. The hepatic cell membrane showed numerous microvilli extending into the Disse spaces. The cytoplasm contained a large amount of granular and a granular endoplasmic reticulum, with areas of continuity between both types. The lipid droplets had different quantities, sizes, and electron densities. Furthermore, lysosomal structures of various sizes, forms, and appearances were dispersed throughout the cytoplasm. The mitochondria were numerous and dispersed all over the cytoplasm with well-developed cristae. The hepatic sinusoids were lined with thin irregular walls, which were made up of endothelial and Von Kupffer cells. The hepatocyte nuclei displayed a typical ultrastructural appearance. The nucleus was round and had a distinct nuclear envelope (Figure 13A). The hepatocyte nuclei in the Harmine mice displayed a typical ultrastructural appearance with a distinct nuclear envelope, consistent with that of the control mice. The hepatic cell membrane showed numerous microvilli emerging into the Disse spaces.

Numerous mitochondria were dispersed throughout the cytoplasm. The cristae on the mitochondria were well-developed and had an oval or spherical shape. Closely spaced, parallel, and flattened cisternae with ribosomes comprised the rough endoplasmic reticulum. Large rosettes or granules of electron-dense glycogen can be easily seen (Figure 13B). Electron microscopic examination of the HFD group demonstrated that the cell membrane of the hepatocytes has flattened microvilli. The cytoplasm exhibits a decrease in intracytoplasmic organelles and glycogen particles with wide empty-looking areas of depleted glycogen. In addition, the cytoplasm has increased fat droplets and many intracytoplasmic vacuoles, like micro and macrovesicular steatosis. A fragmented and rough endoplasmic reticulum and a vesiculated smooth endoplasmic reticulum can be seen dispersed in the cytoplasm.

The mitochondria were either condensed with inconspicuous cristae or swollen with collapsed cristae. Many numbers of lysosomes were found. Hepatocytes expose irregularly shaped nuclei and dense chromatin (Figure 13C). Following the Harmine treatment, the cytoplasmic organelles showed a noticeable improvement under electron microscopy. Areas of glycogen storage are scattered into the cytoplasm. Glycogen is distributed in single or rosette-shaped particles. Lipid droplets were of various numbers, sizes, and electron densities. The liver cells included many mitochondria, and they looked virtually normal. The parallel, flattened cisternae of the well-developed rough endoplasmic reticulum of the liver cells were found to be close to the nuclear envelope. Furthermore, lysosomal structures of various sizes are evident. Hepatocyte nuclei displayed a common ultrastructural appearance with a defined nuclear envelope (Figure 13D).

#### 3.5.2. Pancreas

**Figure 14:**
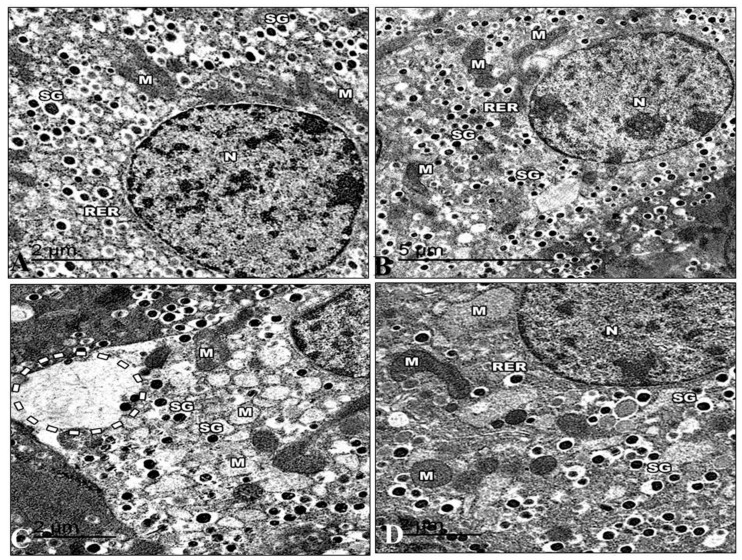
Electron micrographs of pancreatic sections of the control group and other treated groups. (**A**) An electron micrograph of a pancreatic section from the control group revealed a beta cell containing the euchromatic nucleus and a typical nuclear envelope (N). Note that there are mitochondria (M) with apparent cristae and a rough endoplasmic reticulum (RER). Highly crystalloid electron-dense secretory granules (SG) with distinctive halos around them are present. (**B**) An electron micrograph of the Harmine group’s pancreatic section presents a beta cell with a normal nuclear envelope and euchromatic nucleus (N). Its cytoplasm comprises a common rough endoplasmic reticulum (RER) with well-developed cisternae. There are visible cristae with electron-dense matrices in the mitochondria (M). Several secretory granules (SG) with crystalloid electron-dense cores and distinctive halos are visible. (**C**) An electron micrograph of a pancreatic section from the HFD group exhibiting the severe loss of beta cell cytosol, with a marked decrease in cell organelles. The occurrence of wide-empty areas devoid of any cell organelles is evident (dotted circle). Note that the reduction in secretory granules (SG) can be observed; M: Mitochondria. (**D**) An electron micrograph of a pancreatic section from the HFD group treated with the Harmine presenting the beta cells as having a normal nucleus (N) and more euchromatin than heterochromatin. A rough endoplasmic reticulum (RER) and ribosomes are found in the cytoplasm. Some of the mitochondria (M) are still large and have visible cristae. Numerous electron-dense secretory granules (SG) are seen again; however, they appear smaller than in the control group, and their haloes are still invisible. ((**A**); Magnification 20,000×. (**B**); Magnification 12,000×. (**C**); Magnification 20,000×. (**D**); Magnification 20,000×.).

The islets in control mice were big and comprised many beta-cells with mature granules. Their cytoplasm was filled with many electron-dense secretory granules spread throughout the cytoplasm. The mitochondria were numerous and small, with rounded or elongated shapes with electron-dense matrices. Nuclei were round and contained uncondensed chromatin with a regular nuclear envelope. Golgi apparatus can be visible (Figure 14A). The ultrastructure of the Harmine group’s pancreas is displayed in the islets of Langerhans in their normal state. They were formed mainly of beta cells. A rough endoplasmic reticulum containing ribosomes was seen in the cytoplasm. Several Golgi complexes appeared, and the mitochondria had apparent cristae. Beta cells had several secretory granules surrounded by a wide lucent halo diffusely distributed in the cytoplasm. The beta cells had euchromatin nuclei with a regular nuclear envelope (Figure 14B). The electron micrographs of the HFD group revealed that the cytoplasm of the beta cells was now devoid of granules. They had small electron-dense cores with an increased electron lucent halo around them. The mitochondria were distinguished via an extensive loss of matrix and density, and some were ruptured.

Dilated cisterna of the rough endoplasmic reticulum. In the cytoplasm of several of these beta cells, it was possible to see clear dilated and expanded Golgi components. Behind the nuclear membrane, the chromatin seemed compacted and distorted in many beta cell nuclei. (Figure 14C). The electron micrographs of the HFD group that recovered with the Harmine supported the light microscope’s earlier findings. The cytoplasm of the islets contained mitochondria with some dissolution in their cristae. Multiple Golgi complexes were present. The nuclei of the islet cells have ordinary structures. Granules from beta-cells increased in number. The beta cell secretory granules have a regular structure with a dense core surrounded by lucent halo space (Figure 14D).

## 4. Discussion

A strong relationship between obesity and diabetes has been defined. They are a major source of high economic loss due to the high cost of treating many patients. The primary aim of metabolic research is to create novel medications for treating insulin resistance and obesity. Several pharmacological substances and cytokines appear to reduce obesity. Moreover, many bioactive molecules, commonly available as drugs, are extracted from herbs and have strong anti-diabetic properties [20,49,50,51,52].

In the current study, the weight and BMI of HFD mice treated with Harmine were reduced compared to HFD mice. These findings are consistent with those made by Nie et al. [53], who reported that Harmine, when administered at a human-equivalent dose, inhibits body weight gain in obese mice via promoting adipose tissue thermogenesis. The relative organ weights of HFD mice treated with Harmine showed no significant decrease in the pancreas, spleen, and kidney. However, the liver and fat pad weights were decreased in the Harmine-treated HFD mice compared to the HFD mice. This outcome is consistent with other authors, who stated that Harmine-treated mice have less fat content when compared to other groups via blocking the RAC1/MEK/ERK cascade [53].

Furthermore, the blood glucose levels of the HFD mice cured with Harmine were reduced compared to the HFD mice. Waki and his team indicated that Harmine had anti-diabetic properties. When administrated to diabetic mice, it affected the adipocyte gene expression and insulin sensitivity by mimicking the influences of peroxisome proliferator-activated receptor gamma (PPARγ) ligands [27].

The most obvious finding to emerge from the histological sections of the liver of HFD mice is the occurrence of cellular necrosis, swollen Von Kupffer cells, and a large focal area of damaged hepatocytes containing numerous inflammatory cells. Surprisingly, Harmine effectively reduced the hepatic damage and cytoplasmic vacuolations caused by HFD. This result supports other studies linking HFD with inflammation and the necrosis of hepatic cells [54]. When hepatocytes are destroyed, inflammation is generated. Oxidative stress, which is generated by the production of cytokines like tumor necrosis factor-alpha (TNF-α) and Interleukin-1, is the major cause of liver and kidney damage [55]. These improvements in the liver and kidney might be achieved owing to Harmine’s ability to lower the amounts of pro-inflammatory cytokines, including interleukin 6 (IL-6), TNF-α, and interleukine-1 beta (IL-1β).

The kidney of the HFD group showed that most of the renal tubules suffered from severe cellular damage, characterized by the loss of their nuclei or most of their lining epithelial cells. These findings agree with Yu et al. [56], who showed that HFD causes a cascade of clinical alterations in the kidney, involving tubular injury, renal inflammation, and cytokine expression, resulting in kidney disorder. After the administration of Harmine, a reduction in the damage caused to the kidney structure was noticed in comparison with the HFD group. This result may be explained by the fact that Harmine declines the amounts of IL-6, IL-1β, and TNF-α, three pro-inflammatory cytokines.

In general, the pancreatic tissues of mice fed with the HFD had adipocyte and vacuolization caused by triacylglycerol or other lipid metabolites that accumulate in the pancreas [57,58]. After treatment with Harmine, the pancreas returned to its common configuration, with a less pathological appearance and well-defined islet of Langerhans boundaries when compared to the HFD mice. It should be noted that HFD is believed to cause insulin resistance, decrease the function of beta cells in mice, and decrease obesity, which is induced by physical inactivity and chronic high-calorie malnutrition [59]. These results align with those of previous studies by Hull et al. [60]. These findings also confirm those of the previous work, which demonstrated that a high triglycerides content and the presence of ectopic lipids in the pancreas might promote beta cell impairment. Moreover, the chronic administration of 45 and 60% fat diets could cause increased islet size compared to standard chow [61].

The current data found an increase in the diameter and area of pancreatic islets in the HFD group, in parallel with other studies [57,58,62,63,64,65,66]. It is thought that the observed increase in islet area may result from islets compensating for elevated glucose levels and insulin resistance [66]. According to earlier research, the islet hypertrophy that occurs in obese mice may result from hyperinsulinemia, which is a coping mechanism for insulin resistance [67,68].

The electron microscopy of the hepatocytes of the HFD mice revealed many lysosomes of different sizes in the cytoplasm, the aggregation of fat droplets binding to the membrane, flattened endothelial cells, and numerous enlarged Ito cells. These data support evidence from previous observations [69,70,71]. Hepatocytes treated with Harmine showed a considerable improvement in their cytoplasmic organelles. Numerous mitochondria with a nearly standard appearance were seen inside the liver cells, with a well-developed rough endoplasmic reticulum in parallel, flattened cisternae containing ribosomes, and a nucleus with a standard nuclear envelope. This finding was also reported by Friedman et al. [72]. A possible explanation for these results may be that Harmine could enhance the extracellular signal-regulated kinases pathway (ERK) signaling cascade in adipocytes, decline caspase-3 gene expression, which is responsible for programmed cell death in diabetes, and inhibit the nuclear factor-kappa B (NF-κB) signaling pathway [26,53,73].

Another important finding was that the ultrastructure of the pancreas of the HFD mice revealed that some of the nuclei of many beta cells appeared pyknotic, and others were vesicular and had an irregular nuclear envelope. Many of these beta cells had lost the secretory granules of their cytoplasm. The mitochondria were distinguished via a severe loss of matrix and density, and a few were cracked, making it difficult to see their criteria. On the other hand, after treatment with Harmine, the electron micrographs supported the light microscope’s earlier findings. Islet nuclei have typically shaped features. The quantity of beta cell granules increased. This study supports evidence from previous observations [74,75]. These results may be related to Harmine’s inhibition of the DYRK1A or elevated norepinephrine neurotransmitter, which stimulates adipocyte thermogenesis in adipose tissue [76,77].

There were no changes concerning the liver enzyme (ALT) serum levels between the HFD and HFD-treated Harmine groups. This outcome is contrary to that of Diwan [78], who reported that mice administered with *P. harmala* extract showed a remarkable change in the activity of ALT.

This study confirms that there are no significant differences in the creatinine levels between HFD and other treated groups. This outcome contradicts Lu et al. [79], who showed that a high-fat diet produced increased serum creatinine levels. The results for the uric acid levels in this experiment showed a significant increase in diabetic groups compared to other groups. Nitric oxide inhibition and endothelial dysfunction caused by hyperuricemia may eventually result in insulin resistance and diabetes. These results agree with Bhole et al. [80], who mentioned that higher serum uric acid levels raise the chance of developing T2DM over the long term.

After eating a meal rich in fat (55%), the C57BL/6 mice’s insulin levels rose for 6 weeks before dropping off until 20 weeks later. It has been suggested that the eventual reduction occurs because the beta cells fail to compensate for their performance [81]. It is somewhat surprising that the insulin levels in the HFD mice treated with Harmine increased compared to the HFD mice at the end of the experiment. Beta cells overcome insulin resistance by producing adequate insulin, perhaps by increasing their function and mass. However, when there is an increased need for insulin, and the cells fail to respond to this demand, diabetes occurs [65,82].

In the current study, the results for the HbA1c levels in the HFD group showed a significant increase compared to the other groups. These results are similar to studies in which a higher fat intake is associated with higher HbA1c levels and a higher risk of diabetes [83].

The results of the present study reported increased cholesterol levels in HFD mice, similar to previous studies by Vergès [84]. The effect of Harmine on cholesterol levels in our study showed a significant reduction compared to diabetic mice. Triglyceride levels were decreased after the administration of Harmine in HFD mice. This finding contradicts previous studies, which have suggested that high-fat-diet-fed mice did not exhibit rising blood triglyceride levels [85]. These results also match those observed by Kolbus et al. [86], who stated that the triglyceride levels in the HFD mice did not differ between the groups in the fourth week. Harmine can reduce triglyceride serum levels in diabetic rats by controlling PPARγ expression via the inhibition of the Wnt signaling pathway, according to another investigation [27].

On the other hand, there was a remarkable increase in the LDL levels of the HFD mice compared to other groups. These outcomes match the results of Arisue et al. [87], who indicated that serum LDL levels increase in diabetic rats. However, a study by Mollashahi and Kazerani [88] stated that the harmal methanolic extract does not affect serum LDL. There was no difference found between the HFD group and other groups in terms of HDL levels.

The development of chronic diabetes lesions on the arteries, retina, nerves, liver, and kidneys in diabetic people and animals is significantly influenced by cellular oxidative stress [89,90,91]. Several studies support the idea that elevated free fatty acid oxidation and hyperglycemia create oxidative stress due to the liver’s excessive generation of reactive oxygen species, encouraging lipid peroxidation and leading to cell death and structural and functional abnormalities. Under physiological circumstances, the body’s antioxidant defenses counteract the negative effects of these compounds [92,93].

Oxidative stress plays an important role in developing insulin resistance and impairing beta cell function because of its ability to trigger stress-sensitive signals [94]. The results of this study further support the findings of another study by Jin, Xue, Jin, Li and Xu [92], which links oxidative stress with the diabetic state. This study states that MDA can be used as a marker for oxidative stress. The level of MDA in tissues can be used to evaluate the degree of lipid peroxidation. In this research, the concentration of MDA in Harmine-treated mice was considerably lower than in the diabetes groups. In addition, the total antioxidant capacity in Harmine-treated mice was increased compared to diabetic mice. These results coincide with those of previous studies [95].

A possible reason for the anti-diabetic properties of Harmine is that Harmine is an effective DYRK1A that promotes the proliferation of beta cells and enhances blood glucose metabolism [76]. Thus, Harmine might have therapeutic potential in human diabetes therapy. DYRK1A interacts with the receptor tyrosine kinase effector to activate the ERK [96]. Furthermore, other authors have demonstrated that Harmine markedly improves the adipocyte ERK signaling chain [53]. It has several targets, and the related levels of those targets in particular tissues may affect the pharmacological response of the compound in various tissues and cell types. Another explanation for Harmine’s ability to lower blood glucose levels is that it’s essential role in the regulation of PPAR expression, which can improve glucose metabolism in diabetic animals. Harmine works by suppressing the Wnt signaling pathway, like the molecular target of thiazolidinedione anti-diabetic drugs. Therefore, Harmine simulates the impact of PPARγ ligands on the sensitivity to insulin and the regulation of adipocyte genes without exhibiting the negative side effects of thiazolidinedione medications, such as weight gain [27,97]. In obese mice, Harmine treatment delayed the development of diabetes. Mice administered with Harmine showed higher energy expenditure and better insulin and glucose tolerance. This is because of the elevation of blood adiponectin levels and the reduction in lipid and inflammatory profiles [23,27].

## 5. Conclusions

This study has suggested that Harmine has anti-hyperglycemic, weight reduction, and antioxidant properties concerning its therapeutic effects on some renal and liver parameters. Harmine can also reduce the triglyceride and cholesterol serum levels in diabetic mice. There was a significant reduction in the creatinine level with a remarkable improvement in glomerular distension after Harmine administration. Additionally, the hepatocytes partly restored their ordinary configuration. It was noticed that the pathological incidence of damage to the structure of both kidney and pancreas sections was reduced in the Harmine-treated mice compared to the HFD mice. The most obvious finding to emerge from this study is that Harmine can restore the islet’s size and repair the degeneration of islets.

## Data Availability

This manuscript contains all the data related to this study.
